# A Possible Path towards Rapid Development of Live-Attenuated SARS-CoV-2 Vaccines: Plunging into the Natural Pool

**DOI:** 10.3390/biom10101438

**Published:** 2020-10-14

**Authors:** German Todorov, Vladimir N. Uversky

**Affiliations:** 1 Emotional Brain Institute, Nathan Kline Institute, Orangeburg, NY 10962, USA; 2 Department of Molecular Medicine, Morsani College of Medicine, University of South Florida, Tampa, FL 33612, USA; 3 USF Health Byrd Alzheimer’s Research Institute, Morsani College of Medicine, University of South Florida, Tampa, FL 33612, USA

**Keywords:** COVID-19, SARS CoV-2, Vaccine, Treatment strategy, natural live-attenuated vaccine

## Abstract

Severe Acute Respiratory Syndrome Coronavirus 2 (SARS-CoV-2) is the causative agent of the coronavirus disease 2019 (COVID-19) pandemic spreading around the world, causing massive distress to the world’s economy and affecting healthcare systems worldwide. Although some exposed individuals have no symptoms and most symptomatic infections are not severe, COVID-19 cases span a wide spectrum, ranging from mild to critical and sometimes resulting in life-threatening complications, such as pneumonia, severe respiratory distress and cardiac problems. Currently, there is no curative drug for COVID-19 and vaccines are still under development. We are presenting here a strategy for the fast development of natural live-attenuated SARS-CoV-2 vaccines. Our proposed approach is based on screening for, identifying, analyzing and selecting naturally attenuated yet highly immunogenic SARS-CoV-2 strains, which may lead to a shorter cycle of vaccine development, as well as higher vaccine effectiveness.

## 1. Introduction: Towards the Live-Attenuated SARS-CoV-2 Vaccines

The most effective way to prevent the epidemiologic infectious diseases is vaccination, which is successfully used to control measles, mumps, poliomyelitis, and rubella [1], as well as led to the eradication of polio in the USA by 1979. As a result, there are more than 70 vaccines on the market that are targeting approximately 30 infectious agents [2] and helping to prevent morbidity and mortality in millions of people annually [3]. 

Similar to other RNA viruses [4,5], SARS-CoV-2 undergoes rapid inter-species evolution leading not only to the accumulation of mutations that makes new SARS-CoV-2 variants different from the first isolates of the virus obtained in Wuhan (https://www.gisaid.org/epiflu-applications/hcov-19-genomic-epidemiology/), but also to the genetic drift resulting in the attenuation of the virus pathogenicity [5]. Taking these facts into consideration, it was pointed out that “It is entirely plausible that, at present, some individuals, by chance, have already become infected with attenuated versions of SARS-CoV-2” [6]. It was also argued that “knowledge of naturally emerging attenuated SARS-CoV-2 variants across the globe should be of key interest in our fight against the pandemic” [6]. Based on similar premises, we are proposing here a strategy for the rapid development of live-attenuated SARS-CoV-2 vaccines using the targeted search for the attenuated SARS-CoV-2 variants that can serve as the natural source for the live-attenuated vaccines for SARS-CoV-2. 

The use of live-attenuated pathogens is one of the oldest and most effective methods of vaccination. The prominent historic examples of this approach include Edward Jenner’s small pox vaccine based on cowpox virus [7,8] and Albert Sabin’s live-attenuated polio vaccine [9,10].

Evidence is accumulating that SARS-COV-2 infection with the live virus can provide a broad, robust and durable immunity, which, if confirmed, would favor the live-attenuated vaccine approach. An Icelandic study suggested durability of the humoral immune response to SARS-COV-2 infection (live virus), even in older individuals [11]. Other research indicates the development of robust and durable T-cells immunity after SARS-COV-2 infection [12].

In the comprehensive analysis of the molecular basis of coronavirus virulence and vaccine development, it was deemed likely that “the main strategy for producing more efficacious vaccines in the near future will be based on live-attenuated vaccines that lack specific virulence markers” [13]. This conclusion was based on the comparative analysis of various forms of vaccines, such as live-attenuated virus vaccines (i.e., vaccines containing whole pathogens that are designed to replicate within the body inducing mild infection and mild disease symptoms), subunit vaccines that contain only the antigenic parts of the pathogen (e.g., vaccines based on recombinant MERS-CoV Spike (S) protein or its receptor-binding domain (RBD) [14]), replicating recombinant vector vaccines that uses engineered viral vectors with a proven safety record backbone to deliver and express an antigen [15] (e.g., recombinant adenovirus expressing S protein fragments of different size [16,17,18]), and whole-inactivated virus vaccines (i.e., vaccines based on chemically inactivated SARS-CoV virions [19,20,21,22,23] or SARS-CoV-2 virions [24,25]). 

Whereas non-living virus vaccines have some advantages, such as stability and good safety profiles, they are known to cause side effects and can be less efficient than the live-attenuated virus vaccines [13]. On the other hand, live vaccines that use a weakened (or attenuated) form of the pathogen tend to have higher immunogenicity than inactivated vaccines (i.e., those using the killed version of the pathogen) as they imitate natural infection more closely and invoke a wider range of immune responses involving both humoral and cellular immunity. Also, live-attenuated vaccines tend to require fewer administrations to produce reliable and lasting protection. The main known drawback of live-attenuated vaccines is a potential for the reversion to virulence [13]. Nonetheless, all factors considered, a safe and effective live-attenuated SARS-CoV-2 vaccine(s) would clearly be of significant benefit and may have advantages other types of vaccines do not have. The ability to develop live-attenuated SARS-CoV-2 vaccine(s) rapidly would be a valuable tool against the current SARS-CoV-2. Furthermore, intra-species evolution of SARS-CoV-2 potentially leading to future mutations of this virus might cause recurring waves of SARS-CoV-2-related infections resistant to the originally developed vaccine(s). In that case, new vaccines against the novel SARS-CoV-2 variants would again be urgently needed.

Here we propose a possible path towards rapid development of naturally live-attenuated SARS-CoV-2 vaccines. The proposed approach is based on screening for, identifying, analyzing and selecting naturally attenuated yet highly immunogenic SARS-CoV-2 strains, potentially leading to a more rapid cycle of vaccine development, as well as higher vaccine effectiveness.

## 2. The Key Steps of the Proposed Approach

1. Identify populations at the highest risk for severe course of SARS-CoV-2 (e.g., elderly with severe co-morbidities/multiple major risk factors) and find locations of infection clusters in those populations.
2. At or near the location of the infection cluster or simply in very high risk populations (e.g., nursing homes, retirement communities, etc.), screen and test all the highest risk individuals for active SARS-CoV-2 (PCR test).
3. Among those screened, find a subset of individuals with active SARS-CoV-2 injection (positive PCR test), who show no or minimal symptoms and get the samples of the virus from each such person. Then wait till their infection is fully cleared (negative PCR test) and narrow down the selection to those who have not developed any serious symptoms or diagnostic indicators of any long-term health damage associated with COVID-19.
4. In the above group (i.e., high risk individuals who have recovered from SARS-CoV-2 with little or no symptoms/consequences), performs immunological tests and find individuals who have a relatively weak immune system (which would be common in the high risk population you are working with) but still developed a robust immunity against SARS-CoV-2 (preferably both high level of antibodies as well as cellular immunity).
5. Analyze the SARS-CoV-2 variants from the above subgroup of individuals identified in the previous step. Potentially, some of those SARS-CoV-2 variants would be capable of invoking a robust immune response but are sufficiently attenuated as not to cause major symptoms even in the most vulnerable/highest risk individuals. The analysis should include viral genome sequencing and, ideally, tissue cultures tests of infectivity (to verify attenuation and possibly ascertain its mechanism), etc. As an example, one could test for rates of virus binding to ACE2 receptors, for rates of infecting cultured lung cell, etc. Subsequent analyses could also include tests in animal models.
6. The above steps should allow to select SARS-CoV-2 variants with weak infectivity and/or causing low intensity/mild infection. This alone might produce a sufficiently attenuated variant of the virus. Otherwise, one should consider additional steps to further attenuate the virus, such as passaging via human tissue culture while applying selection pressure towards survival of the more attenuated forms of the virus, targeted deletions, etc. The result would be the candidates for the live-attenuated SARS-CoV-2 vaccines.
7. Among the candidate variants of the naturally live-attenuated SARS-CoV-2, identify the ones found in the greatest number of people fitting the above screening criteria. Perform additional screening of all the contacts of the individuals found to have carried naturally SARS-CoV-2 variants. If possible, also screen additional population around the infection cluster under study. Check that the additional individuals found to carry the identified attenuated SARS-CoV-2 variants do not develop a symptomatic infection (or have only mild symptoms) or suffer from any complications. Exclude the live-attenuated SARS-CoV-2 candidates that were found to cause any major issues during this broader screening. The remaining live-attenuated SARS-CoV-2 candidates could be (potentially) suitable for clinical testing.
8. For the most promising live-attenuated SARS-CoV-2 candidate(s) identified in Step 7, keep recursively tracing the contacts of all individuals infected with the candidate strain, thereby (potentially) collecting data on a significant number of individuals infected with it. The goal of this step is to find as many individuals as possible who were infected with, carried, and recovered from the candidate live-attenuated SARS-CoV-2 strain and then to accumulate/analyze enough data to (at least tentatively) determine that the candidate strain: (a) does not cause severe or even moderate infection; (b) is robustly immunogenic; (c) does not revert to a more pathogenic form, etc.
9. Evaluate whether the thus identified naturally live-attenuated SARS-COV-2 candidate(s) is/are suitable for clinical testing as a live-attenuated vaccine. If not, consider additional attenuation steps (e.g., via genetic engineering, such as directed deletion of some specific gene cluster open reading frame(s) [26] or passaging in tissue culture under selection pressure or etc).
10. If the above steps are successful, one would have found a promising live-attenuated SARS-COV-2 vaccine candidate and have accumulated a considerable amount of human clinical data on it. The clinical trials still required to ensure the suitability for vaccination may be less extensive than otherwise, potentially providing a faster path to a safe and effective vaccine.

## 3. Additional Considerations and Concerns

### 3.1. Transmission Rates of the Live-Attenuated Virus

Apparently, in many cases, attenuated virus strains tends to be transmitted less easily (as they tend to reproduce more slowly, be shed less, etc) than the corresponding non-attenuated strains. Natural live-attenuated SARS-CoV-2 strains, if any, may or may not turn out to be easily transmissible. If the candidate attenuated SARS-CoV-2 strain is easily transmissible, we would need to evaluate whether a live-attenuated virus that causes a very mild form of infection but is easy to transmit can be considered sufficiently safe for use as a vaccine, or whether it needs to be further attenuated in the lab, which could slow down the development of the vaccine.

Alternatively, if the candidate attenuated virus stain is not easily transmissible, it may be hard to find (by screening the potentially exposed population) enough people who contracted that particular strain. As a result, it could be difficult to obtain enough of the preliminary human data for that strain, which too could slow down the vaccine development process.

### 3.2. Rates of Natural Evolution of Live-Attenuated Virus

Even if one only screens the subpopulations most likely to carry live-attenuated virus (i.e., asymptomatic and/or mildly symptomatic individuals in the highest risk groups), finding suitable live-attenuated strains may prove slow and/or difficult. That would depend in part on the rate and patterns of natural evolution of the weaker strains, which in turn depends on a variety of factors, such as mutation rate, selection pressures, differential transmission and so forth. RNA viruses tend to have high mutation rates. However, SARS-CoV-2 mutation rate appears to be lower than that of most RNA viruses, possibly due to a “proofreading” enzyme [27]. Also, the evolution of weaker strains tends to be rapid in highly lethal infections with short incubation period. Since SARS-CoV-2 has low mortality rate in the younger and healthier groups as well as a relatively long incubation period, its evolution towards the weaker strains may be relatively slow. On the other hand, the current mutation data on SARS-CoV-2 strains are skewed towards strains obtained from highly symptomatic individuals and may underestimate the prevalence of weaker strains in asymptomatic or mildly symptomatic individuals who never get tested. Furthermore, in fairly isolated high-risk high mortality subpopulations, such as nursing facilities and retirement communities, the evolution patterns of SARS-CoV-2 strains may be closer to those seen in high-mortality infections and favor the emergence of weaker strains more than currently believed. 

All in all, at present there are too many unknowns to predict how much screening of suitable individuals in high-risk subpopulations will be required to find naturally-evolved candidate strains for the development of live-attenuated vaccines. 

### 3.3. Virus Testing

Accurate testing for active SARS-CoV-2 infections, especially eliminating false-positive results, is critical for the success of the proposed approach. False-positives caused by non-viable viral fragments and/or different coronavirus types are not uncommon. Hence, the version of the PCR test with the lowest false-positive rate should be used. It may also be necessary to take multiple samples per patient (from different body sites), use several different PCR primers, correlate PCR test results with antibody tests and T-cell tests, and so forth. Additional confirmation of pre-screened live-attenuated SARS-CoV-2 vaccine candidates would be obtained via culture, full sequencing, passaging and other measures [28,29]. 

### 3.4. Continuous Vaccine Upgrades

If SARS-CoV-2 continues to mutate producing new strains resistant to the original vaccine(s), it may be useful to conduct continuous screening of high risk populations to keep identifying new live-attenuated SARS-CoV-2 candidates to facilitate continuous and timely “upgrading” of the vaccine.

### 3.5. Potential Risks to Look Into

#### 3.5.1. Missed Virulence in the Young

Theoretically, there might arise new strains with low virulence in the old but high in the young. Perhaps that needs to be ruled out explicitly in the vaccine candidate strains.

#### 3.5.2. Reversal of Attenuation

Reversal of live-attenuated virus to more virulent forms is a known (and typically manageable) risk for live-attenuated vaccines. Several examples are given by the design of the genetically stable live-attenuated SARS-CoV vaccines. In the first case, the viral replication fidelity is targeted [30,31]. Replication fidelity of CoVs, which is ~20 times higher than the replication fidelity of other RNA viruses, is mediated by 3′ → 5′ exonuclease (ExoN) that functions in the RNA proofreading [30,31,32,33]. Impairing replication fidelity SARS-CoV by ExoN inactivation generated species that do not revert to virulence, suggesting that ExoN inactivation can be utilized in the stable attenuation of CoVs [31]. In other cases, partial or complete deletion of envelope (E) protein and introduction of mutations in the nonstructural protein 3a (nsp3a) or nsp1 generated attenuated recombinant SARS-CoV viruses that were able to maintain their attenuation after the in vitro and in vivo passages, being capable of fully protecting mice against challenge with the lethal parental virus [34,35,36]. 

#### 3.5.3. Risk of Autoimmune Reactions

In some patients, vaccines can cause autoimmune reactions and there are reported cases of autoimmune diseases that have been correlated with vaccination [2,37]. In fact, despite the fact that vaccination is generally a safe procedure that does not cause serious systemic adverse events, in several studies, single or combined multivaccine procedures were shown to precede development of articular (arthritis, rheumatoid arthritis), autoimmune (systemic lupus erythematosus, diabetes mellitus), and neurological (Guillain Barre syndrome, multiple sclerosis, autism) maladies [37]. However, it seems that live-attenuated vaccines tend to produce fewer autoimmune reactions that non-live vaccines.
